# Supplementary figures and images for: Defining the Syrian hamster as a highly susceptible preclinical model for SARS-CoV-2 infection
Source: Emerg Microbes Infect. 2020 Dec 25;9(1):2673–84. doi: 10.1080/22221751.2020.1858177 (PMC7782266; doi:10.1080/22221751.2020.1858177)

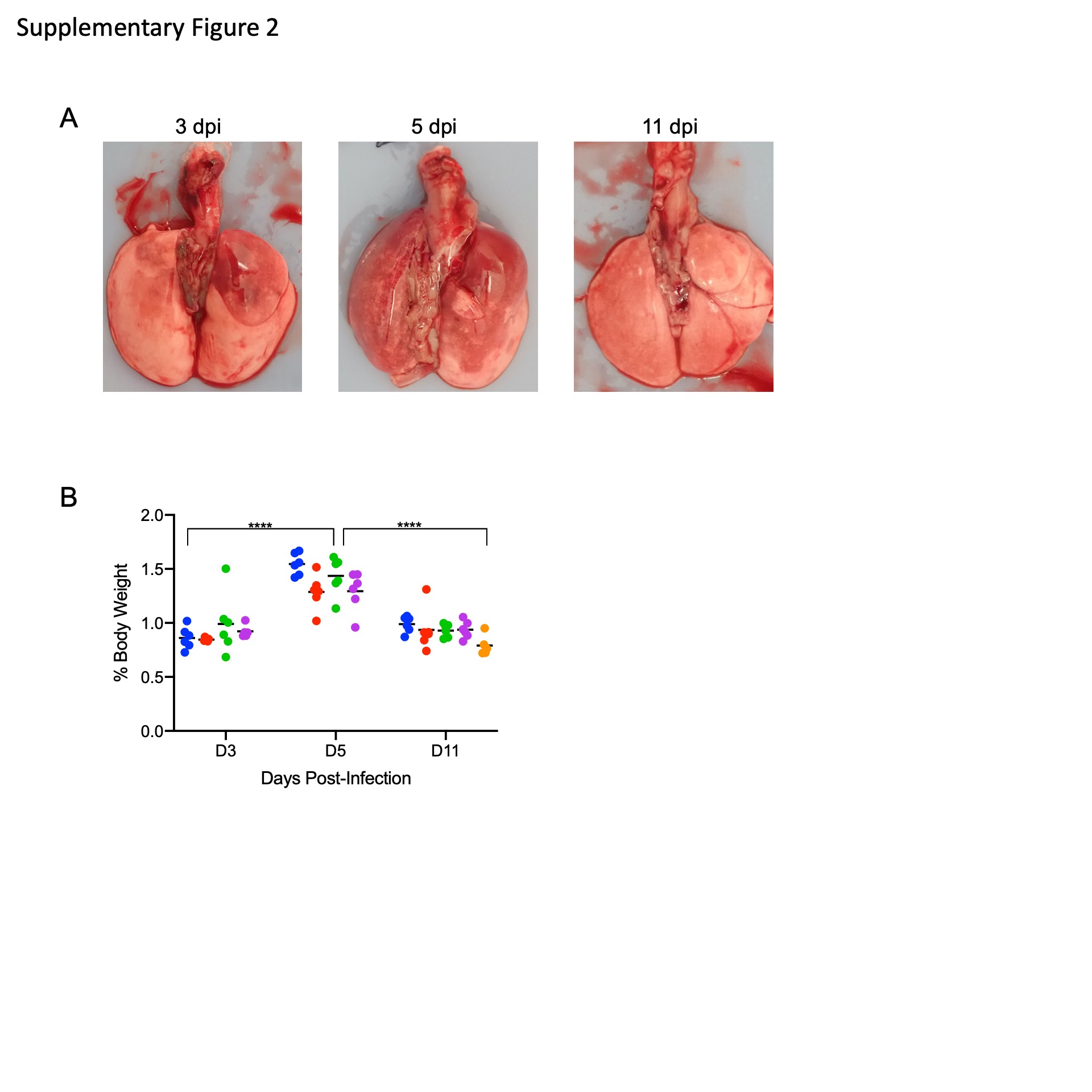

Supplement: Supplemental Material [file TEMI_A_1858177_SM6158.jpg]

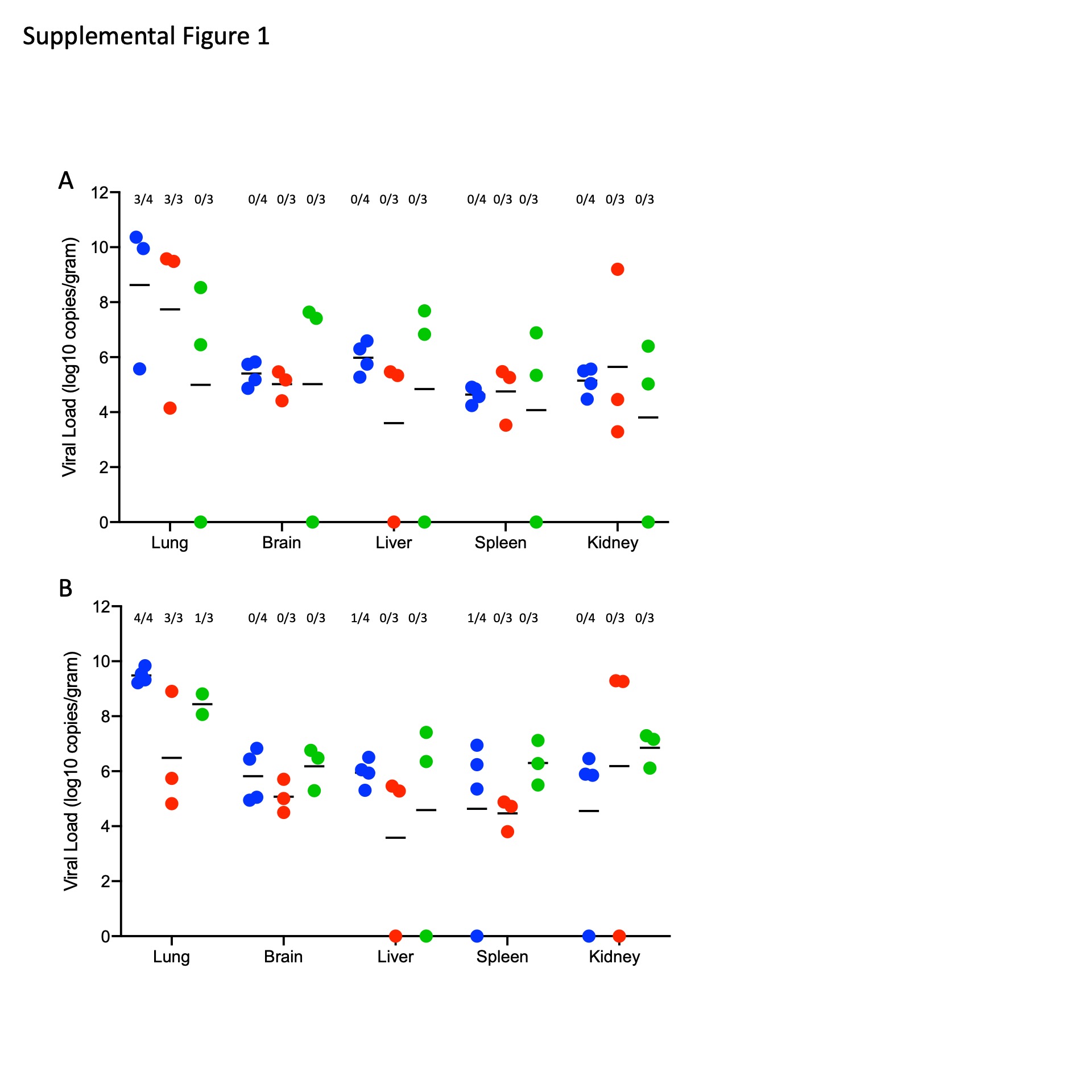

Supplement: Supplemental Material [file TEMI_A_1858177_SM6022.jpg]
